# Expectorant

**Published:** 1893-05

**Authors:** 


### Expectorant.

The best remedy to loosen expectoration is muriate of ammonia combined with some such remedy as syrup of squills. If dry plastic pleurisy be present, iodide of ammonium should be added as follows:

| | | |
|---|---|---|
| ℞ | Ammonii muriat, . . . . | ʒiiss. |
| | Syr. scillæ, . . . . | ʒij. |
| | Ammonii iodidi, . . . . | ʒiss. |
| | Glycerini, . . . . . . | ℥ss. |
| | Syr. prun. virg., q. s. ad., . . | ℥iv. |
| M. | Sig.—ʒj every three hours in water. | |

### Nervous Debility.

| | | |
|---|---|---|
| ℞ | Acid phos. dilut., . . . | ʒiv. |
| | Calisayæ elix., . . . | ℥j. |
| | Elix. valerian ammon., . . | ℥j. |
| | Glycerine, . . . . | ℥ij. |
| | Vini xerici, q. s., . . . | ℥viij. |
| M. | Sig.--Tablespoonful in water three times a day. | |

